# Assessment of Ingested Micro- and Nanoplastic (MNP)-Mediated Genotoxicity in an In Vitro Model of the Small Intestinal Epithelium (SIE)

**DOI:** 10.3390/nano14090807

**Published:** 2024-05-06

**Authors:** Zhenning Yang, Glen M. DeLoid, Joshua Baw, Helmut Zarbl, Philip Demokritou

**Affiliations:** 1 Nanoscience and Advanced Materials Center, Environmental and Occupational Health Sciences Institute, Rutgers University, Piscataway, NJ 08854, USA; zy163@gsbs.rutgers.edu (Z.Y.); gd424@eohsi.rutgers.edu (G.M.D.); 2 Ernest Mario School of Pharmacy, Rutgers University, Piscataway, NJ 08854, USA; jab954@scarletmail.rutgers.edu; 3 Environmental and Occupational Health Sciences Institute, Rutgers University, Piscataway, NJ 08854, USA; hz151@eohsi.rutgers.edu; 4 School of Public Health, Rutgers University, Piscataway, NJ 08854, USA

**Keywords:** micro- and nanoplastics (MNPs), polystyrene (PS), polyethylene (PE), ingestion, genotoxicity

## Abstract

Micro- and nanoplastics (MNPs) have become ubiquitous contaminants of water and foods, resulting in high levels of human ingestion exposure. MNPs have been found in human blood and multiple tissues, suggesting that they are readily absorbed by the gastrointestinal tract (GIT) and widely distributed. Growing toxicological evidence suggests that ingested MNPs may pose a serious health threat. The potential genotoxicity of MNPs, however, remains largely unknown. In this study, genotoxicity of primary and environmentally relevant secondary MNPs was assessed in a triculture small intestinal epithelium (SIE) model using the CometChip assay. Aqueous suspensions of 25 and 1000 nm carboxylated polystyrene spheres (PS25C and PS1KC), and incinerated polyethylene (PEI PM_0.1_) were subjected to simulated GIT digestion to create physiologically relevant exposures (digestas), which were applied to the SIE model at final MNP concentrations of 1, 5, and 20 μg/mL for 24 or 48 h. PS25C and PS1KC induced DNA damage in a time- and concentration-dependent manner. To our knowledge, this is one of the first assessment of MNP genotoxicity in an integrated in vitro ingestion platform including simulated GIT digestion and a triculture SIE model. These findings suggest that ingestion of high concentrations of carboxylated PS MNPs could have serious genotoxic consequences in the SIE.

## 1. Introduction

Plastic waste poses a great threat to the environment due to its rapid production and frequent waste mismanagement. The Organization for Economic Co-operation and Development (OECD) reported that plastic waste is being generated at twice the rate that it was two decades ago, and that most of this waste is released into the environment via landfill disposal, incineration, and waste stream leakage [1]. Plastics have been used in many industrial fields such as health care, agriculture, and food systems, with 40% of plastics currently in use as single use plastics [2]. Moreover, an estimated 22% of plastic waste is not disposed properly, with 11% released into aquatic ecosystems [3].

At least 80% of plastics produced have been and will continue be released or deposited into the aquatic, terrestrial, and atmospheric environments, via wastewater, landfills, and incineration, respectively. Over time, in these environments, the waste plastic will be degraded and fragmented by abrasion, UV damage, thermal stress, and wind and water shear to generate micro- and nanoscale plastic particles and fibers referred to as micro- and nanoplastics (MNPs). MNPs include microplastics, defined as plastic particles or fibers with diameters less than 5 mm, and nanoplastics, which have at least one dimension less than 100 nm [4]. MNPs can also be categorized as either primary or secondary. Primary MNPs are particles and fibers intentionally manufactured with micron or nanoscale dimensions, including microbeads used in personal care products and industrial abrasive applications, and microfibers used in fabrics, all of which are directly released into the environment during use or upon disposal. Secondary MNPs are fragments originating from the breakdown of large plastic debris or primary MNPs via exposure to various degrading processes, including photooxidation resulting from UV radiation (sunlight) in the presence of oxygen, physical abrasion, thermal stress, and biological and chemical degradation [5,6]. Secondary MNPs generated by aging and fragmentation of plastics over time or by municipal incineration of plastic waste are environmental contaminants of increasing concern.

Numerous studies have suggested that MNPs may pose a serious threat to human health, with inhalation and ingestion as primary exposure routes. Examples of inhalation exposures include MNPs released from printing equipment such as laser printers, 3D printers, and photocopiers [7,8], and from incineration of thermoplastic materials at their end of life [9,10,11]. Ingestion is considered one of the most common routes of MNP exposure in humans, as MNPs have been found in foods and drinks or released from plastic food packaging, and have been found to ascend the food chain by trophic transfer [12,13,14,15]. Recent estimates of weekly human MNP ingestion range from 0.1 to 5 g [16]; however, the health consequences of such exposures remain unclear. Growing evidence of ingestion exposures, intestinal uptake and translocation, and bioaccumulation and biodistribution of MNPs in a variety of organisms has been recently reviewed elsewhere [17,18]. Human biomonitoring studies have found significant concentrations of MNPs in human blood [19], urine [20], feces [21], and placenta [22,23], and most recently MNPs were identified in about 58% of atheromas from patients undergoing carotid endarterectomy [24]. In the latter study, the presence of MNPs was associated with a 4.5-fold increased risk of myocardial or cerebral infarction and death [24]. These findings underscore the urgent need for detailed toxicological and human health risk assessment for MNP ingestion exposures.

Evidence of MNP toxicity in a variety of in vitro and in vivo models has been recently reviewed by Ali et al. [25], Yong et al. [26], Yin et al. [27], Wang et al. [28], and Yang et al. [29]. Most studies that have assessed the potential toxicity of MNPs to date have employed commercially available pristine primary spherical polystyrene (PS) and polyethylene (PE) particles [29,30,31,32,33,34]. However, the physicochemical properties (size, shape, surface chemistry, etc.) of such particles are likely to differ substantially from those of environmentally relevant secondary MNPs, which are generated from plastic waste by physical and chemical degradation in the environment and are more representative of real-world MNP exposures. Specifically, photooxidation of plastic particles with a carbon backbone, such as PS, PE, and polypropylene, leads to the formation of new surface functional groups, including carboxyl and carbonyl groups [35]. These surface modifications are likely to result in increased hydrophilicity, which could augment biointeractions, including intestinal uptake, cellular toxicity, and biodistribution. Moreover, environmentally relevant nanosized MNPs tend to aggregate more readily due to their higher attachment efficiencies compared to intentionally manufactured primary MNPs, such as sphere latex MNP models under natural conditions [36]. Hence, it is of great importance to further explore the toxicity and underlying mechanisms of real-to-life MNPs in the environment. Some studies have simulated the production of environmentally relevant MNPs and characterized their physicochemical nature and biological interaction under laboratory conditions, including nanosized polyethylene terephthalate (PET) from the milling of PET pellets and nanosized PET and polyamide (PA) from the cryogenic mechanical fragmentation of related water bottles and fabrics [37,38]. These efforts aim to provide more reliable MNP materials for future MNP toxicity studies.

In our previous study, we found that 25 nm carboxylated PS (PS25C) spheres were readily taken up by and translocated across an in vitro triculture model of the small intestinal epithelium (SIE), and identified the PS MNPs within enterocyte nuclei and apparently in direct contact with a chromosome in a dividing cell [39]. Genotoxicity testing plays an important role in toxicological assessment of all engineered and environmental particles, including MNPs, which is primarily attributed to the irreversible nature and severity of specific health-related adverse consequences that may arise from genotoxic events [40]. Two widely used and traditional genotoxic tests employed for in vitro investigation of chemical genotoxicity are the comet assay and the micronucleus assay [41].

Recent toxicity studies have investigated genotoxic stress and DNA damage caused by MNP particles via the traditional comet assay in different intestinal cell line models, including Caco-2, HT29-MTX, Raji B, and Caco-2/HT29-MTX/Raji B tricultures [42,43,44]. Results from these studies showed that genotoxic effects of MNPs were greatly dependent on MNP physicochemical characteristics and exposure concentrations and durations. However, our understanding of MNP genotoxicity remains limited and incomplete, particularly for low-concentration and long-term exposures to environmentally relevant MNPs that humans might encounter in real-world situations. Moreover, in previous studies of MNP genotoxicity in intestinal models, cells were directly exposed to MNPs suspended in cell culture media, ignoring the role of biotransformations that would occur during human digestion process (e.g., surface chemistry changes and biocorona formation) prior to contact of the MNPs with intestinal cells, which are known to have important impacts on ingested particle biointeractions [45]. In addition, the traditional genotoxicity testing methods employed in these studies have been reported to exhibit low sensitivity and efficacy, as well as inconsistency and susceptibility to user bias [46].

The authors have developed a high-throughput screening platform, CometChip, to measure DNA damage in highly consistent and efficient manner, with minimal user bias and low inter- and intra-experimental noise [47,48]. Our recent studies have applied this technology to assess the genotoxicity of engineered nanoparticles in cells [49]. In this study, we employed the CometChip assay to investigate the potential genotoxicity of selected model MNPs in an in vitro triculture SIE model.

**Figure 1:**
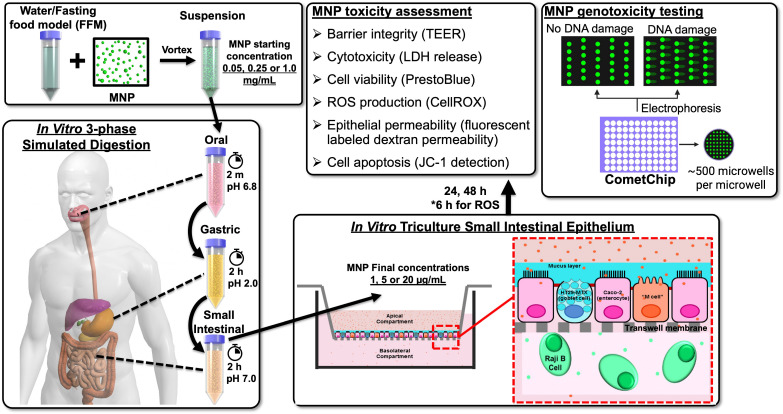
Study design. This figure presents the overall study design, including preparation of a fasting food model (FFM) containing micro- and nanoplastics (MNPs), in vitro three-phase simulated digestion, MNP-containing digestas treatment in the in vitro triculture small intestinal epithelium (SIE) cell model, toxicity assessment and genotoxicity testing of MNPs.

The study design is summarized in Figure 1. Model MNPs studied included PS25C and carboxylated PS microspheres with diameters of 1000 nm (PS1KC), representing primary MNPs, and incinerated PE MNPs with sizes less than 100 nm (PEI PM_0.1_) generated by thermal decomposition of bulk pristine PE pellets using our inhouse integrated incineration exposure platform, representing environmentally relevant secondary MNPs [10]. We reproduced the physicochemical transformations of MNP particles that would occur during digestion in a human gastrointestinal tract (GIT) by subjecting suspensions of tested MNPs in water (fasting food model, FFM) to an in vitro three-phase simulated GIT digestion. An in vitro triculture model, which mimics the SIE, was exposed to the MNP-containing small intestinal digestas (final MNP concentrations in digestas: 1, 5, and 20 μg/mL) for up to 48 h. A panel of cytotoxicity assays was used to assess toxic effects, and the high-throughput CometChip assay was used to measure DNA damage in the triculture model after exposure to MNP digestas.

## 2. Materials and Methods

### 2.1. Particle Preparation, Dispersion and Characterization

#### 2.1.1. Particle Preparation

Primary unlabeled (non-fluorescent) PS25C and PS1KC were purchased from Phosphorex Inc. (Hopkinton, MA, USA) Secondary PEI MNPs were produced by the thermal decomposition of pristine PE plastic pellets using the Integrated Exposure Generation System (INEXS) described in our previous papers [10,50,51,52,53,54].

#### 2.1.2. Particle Dispersion

Due to limited human exposure studies and technical challenges in measuring MNP particles smaller than several microns, to date there are no clear data providing accurate environmentally relevant MNP oral exposure concentrations. A recent report suggested that humans may ingest an average of 5 g of MNPs, roughly equivalent to the weight of a credit card, per week [55]. In our study design, we therefore assumed an average plastic ingestion of 5 g per week and a daily fluid intake of 3 L [56] per person to calculate an average MNP ingestion concentration of approximately 240 µg/mL (5 × 10^6^ µg/(7 d × 3 × 10^3^ mL/d) = 238.1 µg/mL). Based on this estimation, we chose starting oral MNP concentrations of 0.05, 0.25, and 1.0 mg/mL. These concentrations were further diluted through simulated digestion processes and to ensure sufficient nutrition to cells in the in vitro triculture SIE system upon exposure to small intestinal digestas. Stock suspensions of PS25C, PS1KC, and PEI PM_0.1_ MNP particles were dispersed in DI water (representing FFM) and vortexed for 20 s.

#### 2.1.3. Particle Characterization in FFM

Zetasizer Nano ZS90 (Malvern Instruments, Westborough, MA, USA) was used to measure the hydrodynamic diameters (D_h_) and polydispersity index (PdI) of MNPs by dynamic light scattering (DLS) and the zeta (ζ) potentials of MNPs by phase analysis light scattering (PALS). Specifically, MNPs were dispersed in DI water (FFM) at concentrations of 0.05, 0.25, and 1.0 mg/mL, maintained at room temperature, and adjusted to pH ~7.0 by adding NaOH or HCL before measurement.

### 2.2. In Vitro 3-Phase (Oral, Gastric, and Small Intestinal Phases) Simulated Digestions

Suspensions of MNPs in DI water (FFM) were subjected to in vitro simulated 3-phase (oral, gastric, and small intestinal) digestions, as previously described in detail [57]. In brief, oral phase digestas were prepared by combining MNP-FFM suspensions or FFM alone with pre-warmed (37 °C) simulated saliva working solution at a ratio of 1:1 and shaking by hand for 15 s. In the gastric phase, oral phase digestas were mixed with pre-warmed simulated gastric fluid at a ratio of 1:1, followed by a 2 h incubation in a rotary shaking incubator at 37 °C and 100 rpm. The gastric phase digestas were then combined with additional salts and bile extract. The pH of the small intestinal digesta mixture was then adjusted to ~6.99 by adding NaOH or HCL and pancreatic extract (digestive enzymes). The gastric phase digestas was mixed with simulated small intestinal fluid at a ratio of 1:2. The final mixture was incubated for 2 h in a rotary shaking incubator at 37 °C and 100 rpm to imitate the environment of the small intestine. Volumes of 100 IU/mL penicillin and 100 μg/mL streptomycin were added to the final small intestinal digestas to prevent bacterial contamination.

### 2.3. Cell Culture and Triculture SIE Model

Cells were cultured in the incubator maintained at 37 °C + 5% CO_2_. All cell lines used in this study were purchased from Millipore Sigma (Burlington, MA, USA), and cell culture media and supplements were purchased from ThermoFisher Scientific (Waltham, MA, USA). The triculture SIE model was prepared as previously described [57]. Briefly, the in vitro triculture model is developed from Caco-2, HT29-MTX, and Raji B cells. Caco-2 cells differentiate after 2 weeks of culture into absorptive epithelial cells with the characteristic morphological features, markers, and function of small intestinal enterocytes [58,59]. The HT29-MTX cells morphologically and functionally resemble intestinal goblet cells, and provide a mucus layer that coats the triculture epithelium, which acts as a barrier to prevent direct exposure of naked enterocytes to the digesta components and test materials, thus mimicking the luminal environment of the small intestine in vivo [59,60]. The Raji B cells are added as feeder cells to the basolateral compartment to induce the transformation of a small portion (1–3%) of Caco-2 cells to specialized microfold cells (M-cells), which are a crucial component of Peyer’s patches and the lymphoid-associated epithelium of the small intestine, where they provide antigenic surveillance within the SIE model [61].

Caco-2 and HT29-MTX cells were cultured in DMEM with 4.5 g/L glucose and supplemented with required agents, including 10% heat-inactivated fetal bovine serum (FBS), 10 mM HEPES buffer, 100 IU/mL penicillin, 100 μg/mL streptomycin, and 1X non-essential amino acids. Raji B cells were cultured in RPMI media with above required agents. Caco-2 and HT29-MTX cells were trypsinized and resuspended in DMEM media at a density of 3 × 10^5^ cells/mL, respectively, and mixed at a ratio of 3:1. Apical chambers of 6-well 4.7 cm^2^ 0.4 μm pore polycarbonate membrane transwell plates (Corning Inc., Somerville, MA, USA) were seeded with 1.5 mL of the Caco-2/HT29-MTX mixture, and 2.5 mL of complete DMEM media was added to the basolateral chambers. The media was replaced every other day starting from the fourth day after seeding, until the 15th day. On days 15 and 16, the basolateral media was replaced with 2.5 mL of a 1:1 mixture of DMEM and RPMI complete media containing Raji B cells at a density of 1 × 10^6^ cells/mL. 

Caco-2/HT29-MTX cocultures were used to determine oxidative stress. Caco-2 and HT29-MTX were grown at a ratio of 3:1 at a density of 3 × 10^4^ cells/well (100 μL volume) in black-wall, optical bottom plates (Corning Inc., Somerville, MA, USA). The media was changed on the fourth day, then every other day until the 17th day.

### 2.4. Exposure of Triculture SIE to MNP Digestas

Triculture transwell apical media was aspirated and replaced with 2.0 mL small intestinal digestas diluted 1:3 with complete DMEM media or with complete DMEM medium alone (untreated controls), and basolateral media was replaced with 2.5 mL of fresh complete DMEM media. The final MNP concentrations applied to the SIE model were 1, 5, and 20 μg/mL, which resulted from an overall 48× dilution from the corresponding starting oral concentrations of 0.05, 0.25, 1.0 mg/mL. Specifically, the 48× dilution was the result of a 2× dilution in the oral digestion phase, an additional 2× dilution in the gastric phase, another 3× dilution in the small intestinal phase, and a final 4× dilution of the final small intestinal digesta in culture media (2 × 2 × 3 × 4 = 48) prior to treatments in the SIE model. In 96 well plates, media was replaced with 200 µL of small intestinal digestas diluted 1:3 with complete DMEM or complete DMEM media alone. Caco-2/HT29-MTX/Raji B tricultures were incubated for either 24 or 48 h at 37 °C + 5% CO_2_. Caco-2/HT29-MTX cocultures in 96-well plates were incubated for 6 h for assessment of reactive oxygen species (ROS) production.

### 2.5. Determination of Cellular Integrity and Permeability, and Cytotoxicity

#### 2.5.1. Barrier Integrity (Measurement of Transepithelial Electrical Resistance (TEER))

TEER was measured by an EVOM2 Epithelial Volt/Ohm Meter with a Chopstick Electrode Set (World Precision Instruments, Sarasota, FL, USA). Before measurements, the electrode was sterilized with 70% ethanol and placed in DMEM media after air drying in the biosafety cabinet. TEER measurements were conducted during the growth phase immediately before changing media on days 5, 7, 9, 11, 13, 15, and 16, and before and after 24 and 48 h exposures to digestas. The measurements were corrected for blank value (R_Blank_) and transwell filter area (4.67 cm^2^), and were calculated based on the formula: TEER (Ω·cm^2^) = (R − R_Blank_) Ohms × 4.67 cm^2^.

#### 2.5.2. Barrier Permeability (Paracellular-Tight Junction and Transcellular Barrier Integrity)

Fluorescently labeled Alexa Fluor™ 488 3 kDa dextran and Texas Red™ 70 kDa dextran (ThermoFisher Scientific, Waltham, MA, USA) were chosen to assess both paracellular permeability (tight junction integrity, 3 kDa) and transcellular permeability (membrane integrity, 3 kDa and 70 kDa). Both 3 kDa and 70 kDa dextran were dissolved in PBS and vortexed for 30 s to prepare 200 µg/mL stock solutions. Stock solutions were diluted with 1× PBS to 25 µg/mL dextran working solutions. Triton™ X-100 (Electron Microscopy Sciences, Hatfield, PA, USA) diluted with 1× PBS buffer to 0.2% Triton-X100 was employed as the positive control. After 24 and 48 h exposure to digestas or 30 min exposure to the positive control in transwell tricultures, apical and basolateral compartments of transwell plates were washed twice with 3 mL of 1× PBS. A volume of 1 mL of 25 μg/mL fluorescent dextran working solution was then added to the apical compartments and 2 mL of 1× PBS was added to the basolateral compartments of the transwells. The plate was then incubated for 1 h at 37 °C. Basolateral fluids were collected and 200 μL of each were transferred in triplicate wells to a new 96-well plate. Fluorescence was measured at 495 nm (excitation)/519 nm (emission) (Alexa Fluor™ 488 3 kDa dextran) and at 595 nm (excitation)/615 nm (emission) (Texas Red™ 70 kDa dextran) using a SpectraMax M-5 microplate reader (Molecular Devices, San Jose, CA, USA).

#### 2.5.3. Membrane Damage and Cytotoxicity (Lactate Dehydrogenase (LDH) Release)

The CyQUANT^TM^ LDH cytotoxicity assay kit (ThermoFisher Scientific, Waltham, MA, USA) was used to assess cytotoxicity by measuring LDH release into the apical compartment of transwells. Untreated wells (negative controls) were used to determine the spontaneous LDH release from healthy cells. Lysed cells (positive controls) were used as the positive control to determine the maximum LDH release. About 1/10th of the media from the apical compartment(s) of maximum LDH control well(s) was removed and replaced with an equal volume of 10× lysis buffer 45 min before the end of incubation. The reaction mixture was freshly prepared by combining 0.6 mL of assay buffer and 11.4 mL of substrate mix stock solution. Apical media in each well was gently pipetted to mix and 200 μL of apical fluid was transferred to a 1.7 mL tube. All tubes were centrifuged at 10,000× *g* for 5 min at room temperature. A volume of 50 μL of the supernatant from each tube was transferred to each of 3 wells, and 50 μL of the reaction mixture was added to each well in a new 96-well plate. The plate was incubated for 30 min at room temperature and protected from light. A volume of 50 μL stop solution was added and mixed by tapping to terminate the reaction. The absorbance was measured at 490 and 680 nm using the SpectraMax M-5 microplate reader (Molecular Devices, San Jose, CA, USA).

#### 2.5.4. Cell Viability (Mitochondrial Enzyme Activity)

PrestoBlue^TM^ cell viability reagent (ThermoFisher Scientific, Waltham, MA, USA) is a resazurin-based solution (blue dye and weak fluorescence) that readily enter cells, where it can be converted to resorufin compound (red dye and strong fluorescence) via mitochondrial dehydrogenases and reductases in live cells, as a quantitative indicator of cell viability. The PrestoBlue™ viability assay was conducted after 24 and 48 h exposure of cocultures in 96-well plates to MNP-containing digestas. 1 mM staurosporine solution from *Streptomyces* sp. (Millipore Sigma, Burlington, MA, USA) was diluted with DMEM medium w/o phenol red to 0.25 µM staurosporine solution as positive control. Cells were removed from the transwell with 400 μL 0.25% trypsin and centrifuged at 500 rpm for 5 min. Supernatants were removed and cell pellets were resuspended in 1000 μL of completed DMEM as a single cell suspension. A volume of 100 µL of cell suspension was transferred in triplicate wells in a new 96-well plate. A volume of 100 µL of PrestoBlue reagent working solution was added and mixed by tapping the plate. The plate was then incubated for 15 min at 37 °C. Vinblastine (60 nM) was used as the positive control. The fluorescence of resorufin was proportional to the number of viable cells and was measured at 560 nm (excitation)/590 nm(emission) using the SpectraMax M-5 microplate reader (Molecular Devices, San Jose, CA, USA).

#### 2.5.5. Cell Apoptosis (Mitochondrial Membrane Potential (MMP))

JC-1 assay kit (Biotium, Fremont, CA, USA) was performed to determine cellular MMP changes, as a quantitative indicator of cell apoptosis. JC-1 reagent aggregates in the mitochondrial membranes of healthy cells and presents red fluorescence, whereas JC-1 presents green fluorescence as monomeric form in apoptotic and necrotic cells in loss of MMP. The JC-1 cell apoptosis assay was used after 24 or 48 h exposure to MNP-containing digestas. 1 mM staurosporine solution from *Streptomyces* sp. (Millipore Sigma, Burlington, MA, USA) was diluted with DMEM medium w/o phenol red to 0.25 µM staurosporine solution as positive control. 1× Assay Buffer was prepared by diluting 10× Assay buffer in ultrapure water, and JC-1 reagent working solution was prepared by diluting 100× JC-1 reagent in 1× Assay Buffer. A volume of 100 µL of cell suspension was grown in triplicate wells in a new 96-well plate. A volume of 100 µL of JC-1 reagent working solution was added and mixed by tapping the plate. Vinblastine (60 nM) was used as the positive control. Then, the plate was incubated for 15 min at 37 °C. The fluorescence was measured at 550 nm (excitation)/600 nm (emission) (red, mitochondira intact) and 485 nm (excitation)/535 nm (emission) (green, all cells) using the SpectraMax M-5 microplate reader (Molecular Devices, San Jose, CA, USA).

#### 2.5.6. Oxidative Stress (ROS Production)

CellROX^TM^ Green Reagent (ThermoFisher Scientific, Waltham, MA, USA) can be oxidized by cellular ROS and exhibits bright green photostable fluorescence, as a quantitative indicator of oxidative stress. CellROX assay was conducted after 6 h exposure to MNP-containing digestas in the 96-well plate of Caco-2/HT29-MTX cocultures. 10 mM menadione stock solution was diluted with DMEM medium w/o phenol red to 100 µM menadione work solution, 100 µL of which was used to replace 100 µL of medium at 1 h before the end of 6 h exposures as positive control. 5 μM CellROX Reagent working solution was prepared by diluting 2.5 mM stock in DMEM media w/o FBS and w/o phenol red. Treatment media was then removed from the wells, followed by PBS washing, and replaced with 100 μL of CellROX Reagent working solution. The plate was then incubated for 30 min at 37 °C. Cells in the well were then washed twice with PBS after incubation. Menadione (100 µM) was used as the positive control. Fluorescence was measured at 480 nm (excitation)/520 nm (emission) by the SpectraMax M-5 microplate reader (Molecular Devices, San Jose, CA, USA).

### 2.6. Determination of Genotoxic DNA Damage

The total cell number of the remaining cell suspension obtained as described above for the PrestoBlue cell viability assay was counted with a hemocytometer and adjusted to >1.0 × 10^5^ cells/mL. The cell suspension was used to perform the CometChip assay using the CometChip Electrophoresis Starter Kit (Bio-Techne, Minneapolis, MN, USA) according to the manufacturer’s instructions with some modifications. A volume of 100 μL of the cell suspension was loaded into each of 3 wells of a 96-well CometChip, with each well containing approximately 500 microwells. The cells were allowed to settle by gravity into the microwells for 20 min at room temperature. After cell loading, the CometChip was gently washed with 5 mL DPBS 2 to 3 times, then overlaid with low melting point agarose. The cells were then treated with lysis solution for 1 h at 4 °C. DNA unwinding and electrophoresis were performed at 2–8 °C for 50 min at 22 V. The CometChip was then neutralized in 0.4 M Tris–HCl buffer (pH 7.4, Sigma-Aldrich, Burlington, MA, USA) and further equilibrated in 0.02 M Tris–HCl buffer (pH 7.4). Subsequently, the DNA was stained overnight at 4°C with 0.2 × SYBR^®^ Gold (Invitrogen, Carlsbad, CA, USA). Next day, the CometChip was destained with 0.02 M Tris–HCl buffer (pH 7.4) for 1 h at room temperature. The well images were scored using EVOS M5000 Imaging System (Invitrogen). Guicometanalyzer Comet Analysis Software developed in MATLAB (The MathWorks, Natick, MA, USA) was used to score the percentage of DNA in the comet tail (% DNA in Tail) for 200–300 cells from each well of the 96-well CometChip.

### 2.7. Statistical Analysis

Triplicate experiments were performed for TEER, dextran permeability, cytotoxicity, cell viability, cell apoptosis, oxidative stress, and genotoxicity assays. Statistical analysis was performed and graphs were created using Prism software (GraphPad Software, Inc., San Diego, CA, USA). All experimental results were presented as means ± standard deviation of mean (SEM), with SEM being represented by error bars in graphs. Two-way analysis of variance (ANOVA) followed by Tukey multiple comparisons test was used in different groups to analyze statistical differences.

## 3. Results

### 3.1. Characterization of MNP Particles in FFM

The results of size distributions of MNPs in FFM, determined by DLS, are shown in Table 1. The detailed physicochemical characterization of PEI PM_0.1_ MNPs used in this study was presented in our previous study [10,52]. PEI PM_0.1_ comprised a major fraction of organic carbonaceous compounds (99.7% *w*/*w*) and a minor fraction of elemental carbon (0.3% *w*/*w*). Additionally, these PEI particles contained significant levels of both low and high molecular weight polycyclic aromatic hydrocarbons (72.5 μg/g).

### 3.2. In Vitro Cytotoxic Effects of MNPs on the Triculture SIE Model

**Figure 2:**
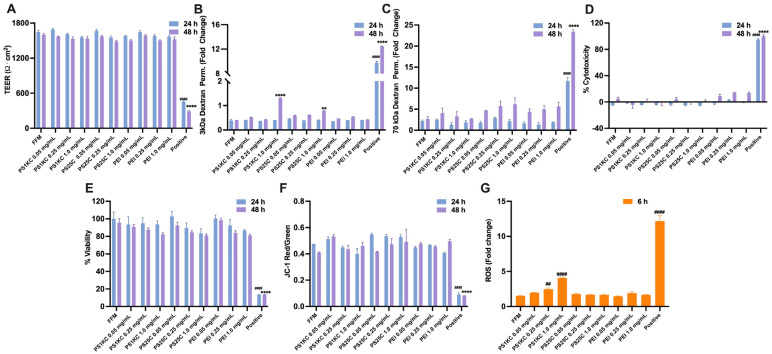
Toxicity assessment of PS25C, PS1KC, and PEI PM_0.1_ MNPs in the triculture SIE model. (**A**) Transepithelial electrical resistance (TEER) after 24 and 48 h exposure. (**B**,**C**) Fold change in apparent permeability coefficient (Papp) assessed with fluorescent labeled 3 and 70 kDa dextran after 24 and 48 h exposure. (**D**) Percent cytotoxicity (percent of LDH release relative to that of lysed control cells) after 24 and 48 h exposure. (**E**) Percent viability (mitochondrial reductase activity relative to untreated control) after 24 and 48 h exposure. (**F**) Cell apoptosis (red/green fluorescence intensity for JC-1 staining) after 24 and 48 h exposure. (**G**) Intracellular ROS levels (fold change relative to untreated control) after 6 h exposure. N = 3–5/group. Initial FFM concentrations are indicated for each material (0.05, 0.25, or 1.0 mg/mL, corresponding to final applied digesta concentrations of 1, 5 and 20 μg/mL). FFM: fasting food model; PS25C: carboxylated PS 25 nm spheres; PS1KC: carboxylated PS 1000 nm spheres; PEI: incinerated polyethylene, PM_0.1_ fraction. Data are presented as mean ± SEM, ** and **** indicate statistical significance at *p* < 0.01 and *p* < 0.0001 compared to the FFM group for 48 h exposure; ## and #### indicate statistical significance at *p* < 0.01 and *p* < 0.0001 compared to the FFM group for 6 h or 24 h exposure.

Results of toxicological assessment of tested MNPs in the triculture SIE model are shown in Figure 2. Both PS25C and PS1KC at the highest “oral” concentration (1.0 mg/mL) significantly increased epithelial permeability to 3 kDa dextran after 48 h exposure, indicating disruption of epithelial tight junction structures (Figure 2B). Neither PS nor PEI at any concentrations significantly altered epithelial permeability to 70 kDa dextran (Figure 2C), indicating that transcellular (plasma membrane) barrier integrity was not impaired.

Exposure to digestas of PS1KC at oral concentrations of 0.25 and 1.0 mg/mL (final concentrations of MNP in digestas: 5 and 20 μg/mL) significantly increased intracellular ROS levels compared to controls after 6 h in a concentration-dependent manner (Figure 2G). No significant MNP-induced changes in barrier integrity (TEER), cytotoxicity (LDH release), cell viability, or cell apoptosis were observed after 24 or 48 h exposures (Figure 2A,D–F).

### 3.3. MNP-Induced DNA Damage Detected Using the CometChip Assay

**Figure 3:**
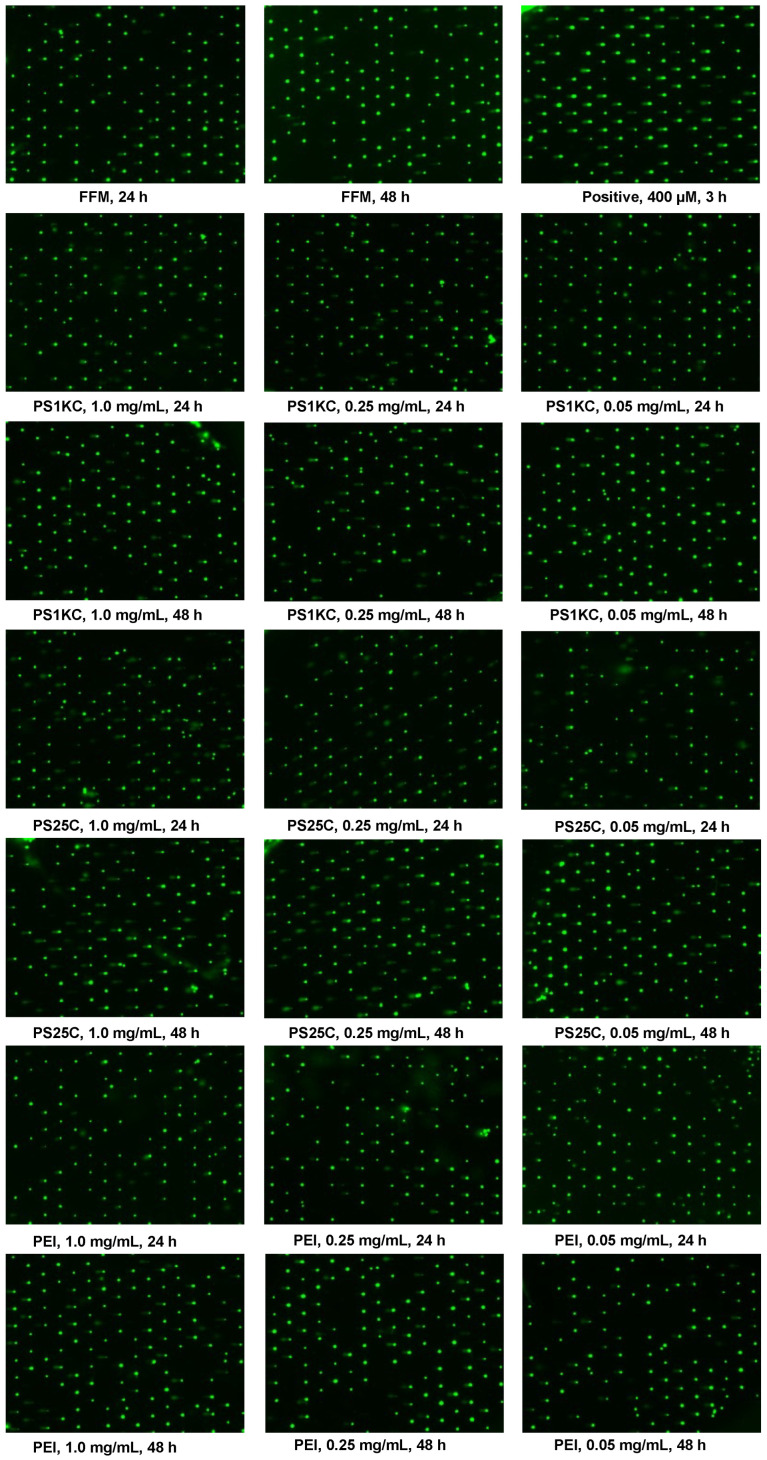
Images of MNP-mediated DNA damage in the triculture SIE model after 24 and 48 h exposure to MNPs using CometChip assay. Positive control cells treated with 400 µM H_2_O_2_ as positive control for 3 h in both 24 and 48 h MNP-treated groups. Magnification: 4×.

Image data depicting MNP-mediated DNA damage in the triculture SIE model using the CometChip assay are shown in Figure 3. The H_2_O_2_-treated positive control group demonstrated significantly larger comet tails, whereas FFM, PS25C, PS1KC, and PEI PM_0.1_-treated groups presented varying increases in comet tail size compared to controls.

**Figure 4:**
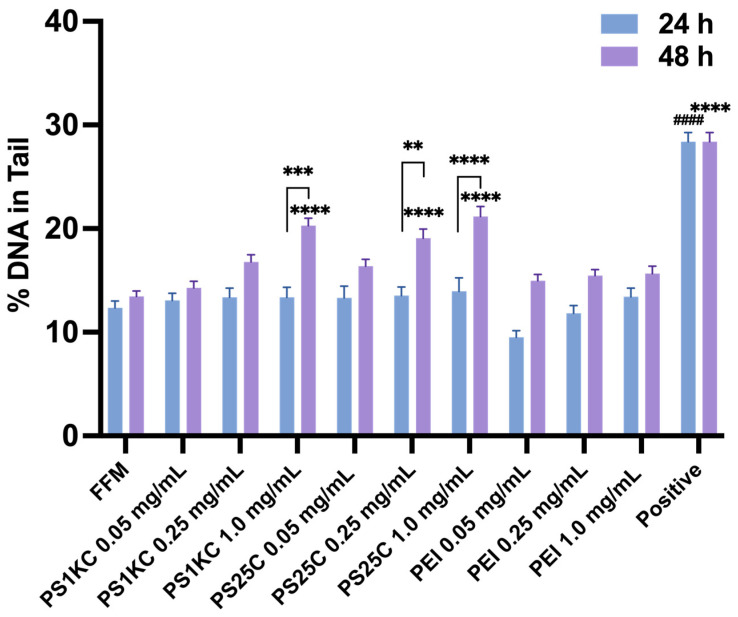
MNP-mediated DNA damage (DNA strand breaks and alkali-labile sites measured as %DNA in Tail) in the triculture SIE model after 24 and 48 h exposure to MNPs. Positive control cells treated with 400 µM H_2_O_2_ as positive control for 3 h in both 24 and 48 h MNP-treated groups. **, *** and **** indicate statistical significance at *p* < 0.01, *p* < 0.001 and *p* < 0.0001 compared to the FFM group or 24 h treated group for 48 h exposure; #### indicate statistical significance at *p* < 0.0001 compared to the FFM group for 24 h exposure.

The % DNA in Tail values, which provide quantitative assessment of DNA damage, including DNA strand breaks and alkali-labile sites, are shown in Figure 4. DNA damage (% DNA in Tail) was significantly increased after 48 h exposures to digestas of 0.25 and 1.0 mg/mL PS25C and of 1.0 mg/mL PS1KC compared to controls and 24 h exposure groups. Moreover, PS25C and PS1KC produced concentration-dependent increases in the % DNA in Tail after 48 h exposure. However, exposures to PEI PM_0.1_ digestas had no significant effects on DNA damage relative to controls at any exposure concentration or duration. Together these results indicate MNP-dependent effects, with exposure to digestas of carboxylated PS but not of PEI MNPs inducing DNA damage in a time- and concentration-dependent manner.

## 4. Discussion

In our previous study, confocal imaging revealed PS25C agglomerates distributed throughout the cytoplasm and within cell nuclei in the triculture SIE model after 24 h exposure, indicating uptake and nuclear translocation of nanoscale PS MNPs [39], suggesting the potential for MNP genotoxicity. In the present study, PS25C and PS1KC at the highest concentration (1.0 mg/mL) significantly increased epithelial permeability to 3 kDa dextran after 48 h exposure, indicating disruption of epithelial tight junction structures (Figure 2B). However, PS1KC and PS25C were found to have no significant effects on transcellular (plasma membrane) barrier integrity and epithelial barrier permeability, cytotoxicity, cell viability, or cell apoptosis after either 24 or 48 h exposure (Figure 2A,B,D–F). These results are consistent with those of previous studies in Caco-2, HT29-MTX, Raji B, or coculture or triculture combinations of these cells [33,39,62], although some studies have reported cytotoxicity of PS MNPs at very high, non-environmentally relevant concentrations. However, in this study we did observe a significant increase in ROS production after 6 h exposure of tricultures to PS1KC at concentrations of 0.25 and 1.0 mg/mL (Figure 2G). In contrast, no significant increase in ROS production was observed after exposure of SIE tricultures to PS25C. This disparity might be due to the relatively faster sedimentation and deposition of the micro-sized PS1KC to the SIE compared to that of the nanosized PS25C. Internalization of sedimented particles may induce mitochondrial dysfunction, leading to activation of the NADPH oxidase system or lysosomal damage, in turn resulting in an excessive release of ROS that exceeds the cellular antioxidant defense capacity, ultimately leading to oxidative stress.

Since, as described above, the majority of MNPs in the environment and to which humans are exposed are secondary MNPs generated through degradation and fragmentation of plastic debris, in this study, we also investigated the effects of a model secondary MNP, PEI PM_0.1_, generated by incineration of pristine PE pellets to mimic the incineration of plastic-containing municipal or medical waste. As with the PS MNPs examined, no significant toxicity was observed after 24 or 48 h exposures to PEI PM_0.1_ MNPs in the triculture model (Figure 2A–F), and as with PS25C, no significant increase in ROS production was observed after 6 h PEI PM_0.1_ exposure (Figure 2G). The absence of ROS responses from PEI PM_0.1_ may, as with PS25C, be in part due to its smaller size relative to PS1KC. In addition, the carboxyl surface modification of PS1KC is likely to result in relatively greater hydrophilicity, which may contribute to augmented biointeractions, including uptake and cellular toxicity. In addition, PE is an aliphatic polymer, lacking the aromatic benzene rings of PS, which may account for the observed differences in reactivity. These differences in physicochemical properties may thus explain the observed size- and polymer-specific toxicity induced by different test MNPs.

DNA damage may occur either through direct interaction with DNA strands or indirectly through oxidative stress caused by ROS generation. Our current understanding of MNP genotoxicity is based on studies that employed the traditional comet assay, the results and interpretation of which are controversial. Significant increases in DNA damage in Raji B cells were previously found using the comet assay after 24 and 48 h exposures to PS MNPs ranging in size from 0.05 to 0.1 μm at concentrations of 25 and 50 μg/mL [44]. This was accompanied by significant increases in oxidative damage after 24 h PS MNP exposures at 50 μg/mL [44]. Similarly, 3 h exposure to 100-300 nm transparent PET MNPs from ground real-life food containers was found to cause modest concentration-dependent increases in DNA strand breaks in Caco-2 and HepG2 cells [43]. Concentration-dependent increases in DNA strand breaks have also been reported in lung A549 cells after 24 h exposures to 136–167 nm ground PET MNPs from food containers [63]. In addition, in our previous in vitro and in vivo inhalation studies with nanoplastics emitted from laser printers, DNA damage, epigenome effects, and effects on DNA repair capacity were found [64,65,66,67]. In contrast, Domenech et al. observed no significant increase in DNA damage or oxidative damage in Caco-2 cells after either short- (24 h) or long-term (8 weeks) exposures to 50 nm PS MNPs [68]. Likewise, no significant DNA damage was seen after 4 h exposures to 50 nm PS at concentrations of 1–50 μg/cm in either Caco-2 or HT29-MTX cell lines [62]. Overall, these results suggest that DNA damage induced by MNPs is highly dependent upon the types and physicochemical characteristics of MNPs, exposure concentrations and durations, and cell types. In addition, the colloidal properties of MNPs in water/digestas may play an important role in MNP toxicity. Nanosized MNPs are more likely to behave as non-settling colloids in the aqueous phase compared to micro-sized MNPs, which may affect their biointeractions and bioaggregation within GIT during digestion, as well as interactions with the intestinal epithelium. The observed moderate but statistically significant differences in hydrodynamic diameter, polydispersity index, and zeta potential of test MNPs might therefore also have played a role in the observed differences in toxicity and genotoxicity between doses in this study. In addition, the plastic colloids could interact with dissolved organic matters in aquatic environments, possibly potentiating their toxicity and exerting indirect effects on human health [69].

In the present study, the significant DNA damage caused by PS25C at 0.25 and 1.0 mg/mL after 48 h exposure (Figure 4) might in part be attributable to their previously demonstrated ability to enter and distribute throughout triculture cells, including nuclei [39]. The significant increase in genotoxic damage levels observed in the triculture cell model after 48 h exposure to PS1KC at the highest concentration (1.0 mg/mL) (Figure 4), on the other hand, might be attributable to the corresponding observed increased intracellular ROS production after 6 h exposure to PS1KC (Figure 2G). No significant DNA damage was observed in the triculture model after either 24 or 48 h exposure to PEI PM_0.1_ MNPs at any concentration. The lack of DNA damage from PEI PM_0.1_ may point to polymer- or other property-specificity in the mechanisms leading to DNA damage. Many of the environmentally relevant MNPs can leach chemical additives directly into the environment or within the body or cells during exposure, which may lead to additional or synergistic genotoxic risks [70]. Additional toxicological studies are needed to identify specific mechanisms involved in various primary and environmentally relevant secondary MNP-induced DNA damage and the roles of MNP properties in those mechanisms.

There is also a need for additional population cohort studies to assess and identify specific potential adverse health outcomes associated with MNPs. Only a few studies to date have identified specific diseases or morbidity associated with MNP exposure. For example, a recent prospective, multicenter, observational study revealed that the presence of MNPs in carotid artery plaques from patients undergoing carotid endarterectomy had a significantly increased risk of a composite of myocardial or cerebral infarction or death compared to patients without MNPs in their artery plaques [24]. In addition, our previous occupational cohort study of workers in the printing industry, where high levels of MNP inhalation exposures take place, revealed significant increases in chronic upper airway and systemic inflammation as well as elevated urinary biomarkers of oxidative damage in healthy photocopier operators [71,72]. These findings underscore the need for more such studies to help us understand and communicate the health risks posed by ongoing and increasing exposures to MNPs.

## 5. Conclusions

In summary, in this study, an in vitro simulated digestion and a triculture SIE model were employed to reproduce physiologically relevant exposures of the SIE to assess the cytotoxicity and genotoxicity of both primary and environmentally relevant secondary MNPs. A high-throughput genotoxicity method, the CometChip assay, was used to determine DNA damage caused by exposures to digestas of the tested MNPs. The results of this study suggest that ingestion exposures to high concentrations of MNPs, specifically PS25C and PS1KC, could have serious genotoxic consequences in the SIE. Although no genotoxicity was observed after exposures to the environmentally relevant secondary PEI PM_0.1_ MNPs, this result cannot be generalized to all environmental MNPs, which can differ in polymer, size, surface chemistry, etc., and thus in any biointeractions with the intestinal epithelium. Additional studies are required to determine toxic and genotoxic effects of both short- and long-term exposures to environmentally relevant secondary MNPs across the full spectrum of polymer types, sizes, and surface chemistries. Further studies are needed to investigate the effects of ingested MNPs in more physiologically relevant experimental models, including humanized cells, organ-on-a-chip, and in vivo animal models. Moreover, additional human MNP exposure studies and data are needed to determine appropriate real-world exposure concentrations and durations for use in future MNP toxicity and genotoxicity studies.

## Figures and Tables

**Table 1:** Colloidal properties of MNPs in FFM. N = 3, values represent mean ± standard deviation.

	Concentration (mg/mL)	Hydrodynamic Diameter (nm)	Polydispersity Index/PdI	z-Potential (mV)
**PS25C**	0.05	35.30 ± 0.25	0.12 ± 0.01	−63.47 ± 2.25
	0.25	35.83 ± 0.35	0.13 ± 0.01	−63.87 ± 0.35
	1.0	33.02 ± 0.01	0.05 ± 0.01	−67.83 ± 0.06
**PS1KC**	0.05	895.87 ± 19.43	0.15 ± 0.07	−48.33 ± 0.45
	0.25	939.60 ± 14.08	0.22 ± 0.15	−48.93 ± 0.61
	1.0	978.97 ± 4.20	0.33 ± 0.07	−50.3 ± 0.17
**PEI PM_0.1_**	0.05	352.40 ± 2.16	0.19 ± 0.01	−37.70 ± 0.17
	0.25	380.60 ± 1.25	0.11 ± 0.01	−40.03 ± 0.32
	1.0	428.10 ± 0.56	0.12 ± 0.02	−40.47 ± 0.32

## Data Availability

Data are contained within the article and Supplementary Materials.

## Supplementary Materials

The following supporting information can be downloaded at: https://www.mdpi.com/article/10.3390/nano14090807/s1, Table S1: Statistical Analysis Results of Colloidal properties of test MNPs in FFM.
