# Supplementary material for: Assessment of Ingested Micro- and Nanoplastic (MNP)-Mediated Genotoxicity in an In Vitro Model of the Small Intestinal Epithelium (SIE)
Source: Nanomaterials (Basel). 2024 May 6;14(9):807. doi: 10.3390/nano14090807 (PMC11085749; doi:10.3390/nano14090807)
Supplement: Supplementary file 1 [file nanomaterials-14-00807-s001.zip › nanomaterials-2969931-supplementary.pdf]

Supplementary Materials

Assessment of Ingested Micro- and Nanoplastic (MNP)-Mediated Genotoxicity in an In Vitro Model of the Small Intestinal Epithelium (SIE)

Zhenning Yang [1,2], Glen M DeLoid [1,3], Joshua Baw [2], Helmut Zarbl [3,4] and Philip Demokritou [1,3,4,*]

[1] Nanoscience and Advanced Materials Center, Environmental and Occupational Health Sciences Institute, Rutgers University, Piscataway, NJ, 08854
[2] Ernest Mario School of Pharmacy, Rutgers University, Piscataway, NJ, 08854
[3] Environmental and Occupational Health Sciences Institute, Rutgers University, Piscataway, NJ, 08854
[4] School of Public Health, Rutgers University, Piscataway, NJ, 08854. zy163@gsbs.rutgers.edu(Z.Y.); gd424@eohsi.rutgers.edu(G.M.D.); jab954@scarletmail.rutgers.edu (J.B.); hz151@eohsi.rutgers.edu (H.Z.)
* Correspondence: philip.demokritou@rutgers.edu

Supplementary Table S1. Statistical Analysis Results of Colloidal properties of test MNPs in FFM.

	**Concentration (mg/mL)**	**PS25C**	**PS1KC**	**PEI $PM_{0.1}$**
**Hydrodynamic diameter** (nm)	0.05 vs. 0.25	ns	$p<0.05$	$p<0.0001$
	0.05 vs. 1.0	$p<0.0001$	$p<0.01$	$p<0.0001$
	0.25 vs. 1.0	$p<0.0001$	$p<0.05$	$p<0.0001$
**Polydispersity Index/PdI**	0.05 vs. 0.25	ns	ns	$p<0.01$
	0.05 vs. 1.0	$p<0.001$	ns	$p<0.01$
	0.25 vs. 1.0	$p<0.001$	ns	ns
**z-potential** (mV)	0.05 vs. 0.25	ns	ns	$p<0.001$
	0.05 vs. 1.0	$p<0.05$	$p<0.01$	$p<0.0001$
	0.25 vs. 1.0	$p<0.05$	$p<0.05$	ns
